# Pan-segmental intraprostatic lesions involving mid-gland and apex of prostate (mid-apical lesions): assessing the true value of extreme apical biopsy cores

**DOI:** 10.1007/s00345-022-04006-2

**Published:** 2022-05-02

**Authors:** Sami-Ramzi Leyh-Bannurah, Svitlana Boiko, Dirk Beyersdorff, Fabian Falkenbach, Jonas Ekrutt, Tobias Maurer, Markus Graefen, Mykyta Kachanov, Lars Budäus

**Affiliations:** 1 Prostate Center Northwest, Department of Urology, Pediatric Urology and Urooncology, St. Antonius Hospital, Gronau, Germany; 2 Martini-Klinik Prostate Cancer Center Hamburg-Eppendorf, Martinistraße 52, 20246 Hamburg, Germany; 3 Department of Diagnostic and Interventional Radiology and Nuclear Medicine, University Medical Center Hamburg-Eppendorf, Hamburg, Germany; 4 Institute of Human Genetics, University Medical Center Hamburg-Eppendorf, Hamburg, Germany

**Keywords:** Prostate cancer, mpMRI, Image-guided biopsy

## Abstract

**Objective:**

When considering increased morbidity of apical biopsies, the added diagnostic value of separate targeting of mid-gland and apical segment of the pan-segmental mid-apical mpMRI prostate cancer (PCa) suspicious lesions was assessed.

**Materials and methods:**

A total of 420 patients with a single mpMRI PCa-suspicious PI-RADS ≥ 3 intraprostatic lesion extending from the mid-gland to the apical segment of the gland underwent transrectal MRI-targeted (TBx) and systematic prostate biopsy. Clinically significant PCa (CsPCa) was defined as Gleason Score (GS) ≥ 3 + 4. PCa detection rates of TBx cores were assessed according to targeted anatomical segments. Finally, the diagnostic values of two theoretical TBx protocols utilizing 1-core (A) vs. 2-cores (B) per anatomical segment were compared.

**Results:**

TBx within the pan-segmental mid-apical lesions yielded 44% of csPCa. After stratification into mid- vs. apical segment of the lesion, csPCa was detected in 36% (mid-gland) and 32% (apex), respectively. Within the patients who had no csPCa detection by mid-gland sampling (64%, *n* = 270), extreme apical TBx yielded additional 8.1% of csPCa. Comparison of extreme apical TBx strategy B vs. overall PCa detection in our cohort revealed corresponding similar rates of 49 vs.50% and 31 vs.32%, respectively.

**Conclusion:**

Separate analyses of both segments, mid-gland and apex, clearly revealed the diagnostic contribution of apical TBx. Our findings strongly suggest to perform extreme apical TBx even within pan-segmental lesions. Moreover, our results indicate that a higher number of cores sampled from the mid-gland segment might be avoided if complemented with a two-core extreme apical TBx.

**Supplementary Information:**

The online version contains supplementary material available at 10.1007/s00345-022-04006-2.

## Introduction

Systematic transrectal ultrasound-guided biopsy of prostate (SBx) is still widely used for primary prostate cancer (PCa) diagnosis [1]. In comparison, multi-parametric magnetic resonance imaging (mpMRI) of the prostate and image-guided targeted biopsy (TBx) is proven to be of superior diagnostic accuracy [2, 3].

Recent studies on TBx sampling techniques focused on both: optimization of targeting accuracy and minimizing the sampling heterogeneity, e.g., based on a restriction to a minimum number of TBx cores needed for clinical significant PCa (csPCa) detection [4–7]. However, most series did not account for technical and anatomical limitations of biopsy techniques utilized, e.g., transrectal approach, such as limited ability to sample apical or anterior lesions [8, 9].

With regard to apical PCa lesions, none of the related series adjusted for either the anatomical site of biopsies nor the number of TBx cores sampled [10–12]. Especially, the proximity to the sphincter region and the number of cores taken might contribute to the morbidity of the biopsy [13, 14].

Pan-segmental mid-apical mpMRI PCa-suspicious lesions are more prevalent than clearly delimited, exclusively apex-located lesions [9]. In this clinical scenario, the assessment of the true diagnostic value of anatomic segment of mpMRI lesion may allow further tailoring and individualizing of the TBx strategy, e.g., adjustment of the number of biopsy cores taken. Such an approach, like, e.g., sparing the extreme apex in pan-segmental mid-apical lesion can result in less-invasive procedures, reducing the morbidity of transrectal and perineal anatomic approaches.

Therefore, our study aims to explore whether extreme apical TBx sampling is of added diagnostic value for csPCa detection in men with mid-apical PCa-suspicious mpMRI lesions. Moreover, we propose an optimized TBx sampling strategy in men with aforementioned clinically important mpMRI lesions.

## Materials and methods

### Patient selection

Overall, we identified 420 patients, harboring a single mpMRI PCa-suspicious intraprostatic lesion extending from the mid-gland to the apical segment of the gland. All patients underwent MRI/ultrasound fusion-guided TBx combined with ≥ 8-core SBx in the Martini-Klinik Prostate Cancer Center Hamburg-Eppendorf, Germany, from 2015 to 2021.

The indication for mpMRI was based on clinical suspicion of PCa and initiated by the referring physician. Inclusion criteria for TBx consisted of at least one PCa-suspicious mpMRI lesion with a PI-RADS v2 score ≥ 3 [15]. The pretreatment PSA level was measured before digital-rectal examination and TRUS/biopsy session. Clinical stage was assigned by the attending urologist according to the 2002 TNM system [1].

All patients had no history of prior mpMRI or TBx. All data were prospectively recorded in an institutional review board-approved database. Written informed consent for retrospective data analyses was signed by all patients.

### mpMRI protocol

MpMRI imaging was performed according to the 2012 European Society of Urogenital Radiology (ESUR) guidelines as also PI-RADS v2 guideline recommendations and contained T2-weighted imaging sequences, diffusion weighted imaging sequences, and dynamic gadolinium-based contrast-enhanced imaging sequences [16]. In-house 3.0T MRI scans (Ingenia, Philips Medical Systems, Best, The Netherlands) were performed using a phased-array coil, according to the previously described protocol [17, 18]. All mpMRI were read by dedicated uro-radiologist with > 20 years of experience, who had access to clinical data. All mpMRIs initially conducted by third-party radiologist later received a second reading in our institution. All mpMRI examinations were reported according to PI-RADS v2 guideline recommendations [15]. The number of lesions, as well as the corresponding anatomical segment, i.e., region of interest (ROI), were defined according to the PI-RADS v2 segmentation model [15]. Specifically, we used the division between prostate base, mid-gland apex, i.e., upper, middle, and lower third of the prostate, respectively. Pan-segmental mid-apical lesions were defined as those affecting the both: apical and mid-gland segments (henceforth referred to as mid-apical) [15].

### MRI/ultrasound fusion-guided targeted biopsy

All patients received an oral antibiotic prophylaxis in accordance with European Association of Urology (EAU) Guidelines [1]. Transrectal biopsy was performed under peri-prostatic regional anesthesia with bupivacaine. TBx combined with SBx was performed with Urostation (Koelis, La Tronche, France). All biopsy cores were sampled and documented separately. Histopathological biopsy interpretation was performed by dedicated uropathologists for all specimens. Clinically significant PCa (csPCa) was defined as Gleason score ≥ 3 + 4 [19].

### Statistical analyses

Within the patients included, TBx PCa detection rates were compared after stratification according to targeted anatomical segments: (a) overall PCa detected in all targeted segments vs. (b) in the mid-gland segment vs. (c) in the apical segment (henceforth referred to as extreme apical targeting). Latter served to estimate the added value of extreme apical lesion targeting.

To account for the effect of number of TBx cores sampled per mid-gland vs. apical segments, analyses were stratified according to respective number of TBx cores sampled per segment. Specifically, we examined following theoretical biopsy strategies: (1) First TBx core mid-gland and second TBx core apical (henceforth referred to as biopsy strategy A), (2) First and second TBx cores mid-gland and third and fourth TBx cores apical (henceforth referred to as biopsy strategy B). Latter biopsy strategy was restricted to 372 patients with sufficient number of at least two cores sampled per anatomical segment.

Descriptive statistics included frequencies and proportions for categorical variables and median and interquartile ranges (IQR) for continuously coded variables, respectively. Chi-square test was used for categorical variables and *t* test for continuously coded variables. All tests were two-sided with *p* values < 0.05 to indicate statistical significance. Analyses were performed using the statistical package for R (the R foundation for Statistical Computing, version 3.6.3).

## Results

In patients harboring pan-segmental mid-apical MRI lesions (*n* = 420), median age and PSA were 66 (IQR 61–71) years and 7.7 (IQR 5.3–10.8) ng/ml, respectively (Table 1).

**Table 1:** Descriptive characteristics of the patients (*n* = 420) harboring pan-segmental mid-apical mpMRI lesion

Baseline characteristics	Patients with mid-apical mpMRI lesion (*n* = 420)	
Age, years (median, IQR)	66	61–71
PSA, ng/mL (median, IQR)	7.7	5.3–10.8
Number of prior negative systematic biopsy sessions (*n*, %):		
Naïve	217	52%
1	134	32%
≥ 2	69	16%
Maximum PI-RADS score (*n*, %):		
3	126	30%
4	240	57%
5	54	13%
Overall number of biopsy cores (median, IQR)	14	13–16
Number of targeted biopsy cores (median, IQR)	7	6–9
Number of targeted biopsy cores from the mid-gland segment (median, IQR)	4	3–6
Number of targeted biopsy cores from the apical segment (median, IQR)	3	2–4
Targeted biopsy highest Gleason score (*n*, %)		
No tumor	156	37%
3 + 3	80	19%
3 + 4	97	23%
4 + 3	31	7.4%
≥ 4 + 4	56	13%

*IQR* interquartile range; *mpMRI* multi-parametric magnetic resonance imaging

The maximum PI-RADS score of 3, 4, and 5 was found in 126 (30%), 240 (57%), and 54 (13%) of the patients. A median number of 7 (IQR 6–9) TBx cores were sampled per lesion. TBx findings yield 44% (*n* = 184) of csPCa, 19% (*n* = 80) of GS 3 + 3, and 37% (*n* = 184) of no PCa.

First, analyses focused on the TBx detection rates stratified according to either mid-gland or apical segments (Table 2). Specifically, a median of 4 (IQR 3–6) cores were exclusively sampled from the mid-gland segments, whereas a median of 3 (IQR 2–4) cores were sampled from the apical segments.

**Table 2:** Comparison of targeted biopsy highest Gleason scores stratified according to targeted anatomical segments in the patients with pan-segmental mid-apical PCa-suspicious mpMRI lesion

Overall PCa detection within the cohort (*n* = 420)	Location of targeted biopsy cores within mid-apical mpMRI lesion					
Targeted biopsy highest Gleason score (*n*, %):	Overall (mid-gland and apex)		Mid-gland		Apex	
(a)						
No tumor	156	37%	199	47%	212	50%
3 + 3	80	19%	71	17%	75	18%
3 + 4	97	23%	89	21%	78	19%
4 + 3	31	7.4%	22	5.2%	23	5.5%
≥ 4 + 4	56	13%	39	9.3%	32	7.6%

Biopsy strategy A (*n* = 420)	Location of targeted biopsy cores within mid-apical mpMRI lesion					
Targeted biopsy highest Gleason score (*n*, %)	Overall (mid-gland and apex)		Mid-gland		Apex	
(b)						
No tumor	214	51%	281	67%	263	63%
3 + 3	76	18%	53	13%	57	14%
3 + 4	82	20%	56	13%	67	16%
4 + 3	14	3.3%	11	2.6%	10	2.4%
≥ 4 + 4	34	8.1%	19	4.5%	23	5.5%

Biopsy strategy B ( *n* = 372)	Location of targeted biopsy cores within mid-apical mpMRI lesion					
Targeted biopsy highest Gleason score (n, %):	Overall (mid-gland and apex)		Mid-gland		Apex	
(c)						
No tumor	150	40%	215	58%	190	51%
3 + 3	77	21%	57	15%	67	18%
3 + 4	83	22%	60	16%	73	20%
4 + 3	19	5.1%	14	3.8%	15	4.0%
≥ 4 + 4	43	12%	26	7.0%	27	7.3%

*PCa* prostate cancer; *mpMRI* multi-parametric magnetic resonance imaging

Further stratification according to the theoretical biopsy protocol implemented: (a) Overall PCa detection within the cohort (*n* = 420); (b) biopsy strategy A—first targeted biopsy core mid-gland and second core apical (*n* = 420); (c) biopsy strategy B—first and second targeted biopsy cores mid-gland and third and fourth cores apical (*n* = 372)

Mid-gland TBx sampling did not detect any PCa in 47% (*n* = 199), and separate apical TBx sampling similarly did not detect any PCa in 50% (*n* = 212). However, the added extreme apical TBx sampling decreased the non-detection rate by 10%, i.e., down to 37%. The mid-gland TBx sampling detected GS 3 + 3 in 17% (*n* = 71), separate apical TBx sampling detected GS 3 + 3 in 18% (*n* = 75), and the added extreme apical TBx sampling yielded a virtually identical rate of 19%. The corresponding rates for csPCa were 36% (*n* = 150) and 32% (*n* = 133), respectively. Here, within those patients that had no csPCa detection by mid-gland sampling (64%, *n* = 270), additional extreme apical targeting yielded additional 8.1% csPCa. Conversely, within those patients that had no csPCa detection by separate apical sampling, mid-gland sampling yielded additional 12.5% of csPCa.

Second, stratification according to the two different theoretical biopsy protocols revealed following findings. At biopsy strategy A, in which the mid-gland segment is sampled first with a single core, followed by a second core of the apical segment, the added extreme apical sampling improved the rates of ≥ GS 3 + 3 from 33% to 49% and of csPCa from 20% to 31%, respectively. Specifically, within those patients that had no csPCa detection by one single core mid-gland sampling (i.e., either no tumor detection or GS 3 + 3), extreme apical targeting with one targeted biopsy core yielded additional 11% of csPCa.

At biopsy strategy B, in which the mid-gland segment is sampled first with two consecutive cores, followed by two further cores of the apical segment, the added extreme apical sampling improved the rates of ≥ GS 3 + 3 from 42% to 60% and of csPCa from 27% to 39%, respectively. Specifically, within those patients that had no csPCa detection by two-core mid-gland sampling, extreme apical targeting with two targeted biopsy cores yielded additional 12% of csPCa.

Finally, PCa and csPCa detection rates of biopsy strategy B approximate those of the overall PCa detection in our cohort (i.e. before any stratification according to number of cores sampled), with rates of 60% vs. 63% and 39% vs.44%, respectively. Anatomical stratification reveals that differences are mainly driven by targeting the mid-gland segment, 42% vs. 52% and 27% vs. 36%, respectively. In contrast, comparison of extreme apical targeting of biopsy strategy B vs. overall PCa detection in our cohort revealed corresponding, similar rates of 49% vs. 50% and 31% vs. 32%, respectively.

## Discussion

The finding of exclusively apical PCa lesions is quite rare, particularly compared to the rather frequently reported combination of pan-segmental lesions that extend from mid-gland to the prostate apex [9, 20]. Such extent of mid-apical lesions are considered to be challenging, especially in case of apical or anterior targeting utilizing the transrectal biopsy approach [21–23]. Moreover, anatomical proximity to the apical urethra might be associated with greater morbidity [13, 14, 19, 20, 24, 25]. Despite such important implications of specific targeted anatomical segments, these considerations were not accounted for in large studies examining biopsy strategies with focus on sufficient number of cores per target.

Specifically, three contemporary prospective trials proposed a specific number of cores per target within TBx: four in the PRECISION trial vs. three cores in the MRI-FIRST trial vs. two to four cores in 4M trial [2, 26, 27]. These findings resulted in the European Association of Urology Guidelines recommendation to obtain a higher number of three to five biopsy cores per target, compared to the former recommendation of only two cores per target in the 2016 consensus statement by the American Urological Association (AUA) and Society of Abdominal Radiology (SAR). The intended effect is to reduce the risk of missing PCa or undersampling the lesion [1, 2, 4, 26–28]. Therefore, we examined whether extreme apical targeted biopsy sampling in men with mid-apical PCa-suspicious mpMRI intraprostatic lesions are of added value for csPCa detection and propose an optimized TBx targeting and sampling strategy in men based on clinically important primary mpMRI imaging.

Our study had several important findings. First, anatomical stratification of the lesions according to mid-gland and the apical and segments demonstrated heterogeneous TBx PCa findings, such as higher PCa and csPCa detection in the mid-gland lesion segment 53% and 36% vs. 50% and 32% in the apex, respectively. These findings support the recommendation of the European Association of Urology Guidelines to obtain a higher number of TBx cores to avoid heterogeneity. However, even despite higher numbers of TBx sampled per segment: a median of 4 (IQR 3–6) from the mid-gland segment vs. a median of 3 (IQR 2–4) does not overcome the observation of intraprostatic heterogeneity of biopsy yield.

Second, aforementioned stratification according to the anatomical segments enabled to assess a potential added value of the extreme apical targeting. A substantial additional yield of 8.1% csPCa of the apical TBx demonstrates that extreme apical targeting should not be spared from an oncological standpoint [13, 14, 19, 20, 24, 25], even after considering transrectal limitations of apical targeting and associated potential complications due to the proximity of the urethra. In consequence, particularly at such apical targeting, the optimum number of cores should be considered to provide oncological safety as well as to reduce biopsy-related morbidity.

Third, further stratification according to the two theoretical biopsy strategies A and B, i.e., utilizing two vs. four TBx cores per lesion or one vs. two TBx cores per anatomical segment, represents a real-world application of the consensus statement recommendations by AUA and SAR, which propose a minimum number of two TBx cores per lesion, vs. PRESICION trial, which applied a four TBx cores per lesion protocol [2, 28]. Expectedly, we observed an up to 12% higher PCa yield per segment or both, mid-gland and apex, combined if at least two cores were sampled per anatomical segment compared to only one core per segment. However, compared to the unstratified overall cohort with respective median number of cores of 4 (IQR 3–6) and 3 (IQR 2–4) for mid-gland and apical segments, respectively, we observed that the apical sampling appeared to be saturated after already two cores. This finding emphasizes that the originally higher number of TBx cores used for the PCa detection within the apical segment of mid-apical lesions could be optimized. Reduction of the number of apical cores likely translates to a more favorable morbidity profile, i.e., fewer complications [13, 14]. Additionally, the known association between apical shapes and voiding symptoms should be taken into the account before biopsy session [24]. Conversely, the mid-gland lesion targeting yield indicates that a PCa detection based on two targeted biopsy cores can be further improved by relying on further core sampling within the same anatomical segment.

As an alternative solution in this scenario, our results clearly demonstrate that a higher number of cores sampled from the mid-gland segment might be avoided if the biopsy strategy is complemented with a two-core extreme apical targeting.

Our study is not devoid of limitations. First, radical prostatectomy results were not available as reference standard in the majority of patients. Second, it is important to acknowledge the explorative study design, which precludes generalizability in general clinical practice. Our study addresses only one particular type of mpMRI lesion extension (mid-apical mpMRI lesions), and thus, our results are not representative and could not be transferred to the lesions of other locations. Third, the results of our study, as well as proposed biopsy protocols apply for the transrectal biopsy technique and cannot necessarily be transferred to a transperineal biopsy technique. Finally, our data originate from a single tertiary referral center, with specific patient characteristics and involvement of a highly experienced genitourinary radiologist and pathologist, which might lead to limited comparability and generalizability of our findings between institutions.

## Conclusions

To our knowledge, this series is the first to address the TBx sampling strategy of pan-segmental mid-apical mpMRI lesion. We demonstrate that sampling of both segments in patients with mid-apical lesions avoids undersampling. Moreover, our findings suggest that higher number of TBx cores sampled from the mid-gland segment might be avoided if the biopsy strategy is complemented with two extreme apical TBx cores. However, sparing of extreme apical TBx sampling to avoid proximity to the sphincter resulted in decreased diagnostic accuracy.

## Supplementary Information

Below is the link to the electronic supplementary material.

Supplementary file1 (DOCX 469 KB)

## Abbreviations

MRI: Magnetic resonance imaging
mpMRI: Multi-parametric magnetic resonance imaging
SBx: Systematic transrectal ultrasound-guided biopsy
TBx: Targeted biopsy
PCa: Prostate cancer
csPCa: Clinical significant prostate cancer
GS: Gleason score
PI-RADS: Prostate imaging reporting and data system
IQR: Interquartile ranges

## References

[1] Mottet N, Conford P, van den Bergh RCN et al (2021) EAU-EANM-ESTRO-ESUR-ISUP-SIOG guidelines on prostate cancer 2021. In: EAU guidelines. Presented at the EAU annual congress Milan 2021. European Association of Urology Guidelines Office, Arnhem, The Netherlands

[2] Kasivisvanathan V, Rannikko AS, Borghi M (2018). MRI-targeted or standard biopsy for prostate-cancer diagnosis. N Engl J Med.

[3] Ahmed HU, El-Shater Bosaily A, Brown LC (2017). Diagnostic accuracy of multi-parametric MRI and TRUS biopsy in prostate cancer (PROMIS): a paired validating confirmatory study. Lancet.

[4] Leyh-Bannurah SR, Kachanov M, Beyersdorff D (2020). Minimum magnetic resonance imaging-ultrasound fusion targeted biopsy cores needed for prostate cancer detection: multivariable retrospective, lesion based analyses of patients treated with radical prostatectomy. J Urol.

[5] Calio BP, Sidana A, Sugano D (2018). Risk of upgrading from prostate biopsy to radical prostatectomy pathology-does saturation biopsy of index lesion during multiparametric magnetic resonance imaging-transrectal ultrasound fusion biopsy help?. J Urol.

[6] Porpiglia F, De Luca S, Passera R (2017). Multiparametric magnetic resonance/ultrasound fusion prostate biopsy: number and spatial distribution of cores for better index tumor detection and characterization. J Urol.

[7] Kachanov M, Leyh-Bannurah SR, Roberts MJ (2021). Optimizing combined magnetic resonance imaging-targeted and systematic biopsy strategies: sparing the multiparametric magnetic resonance imaging-negative transitional zone in presence of exclusively peripheral multiparametric magnetic resonance imaging-suspect lesions. J Urol.

[8] Taira AV, Merrick GS, Galbreath RW (2010). Performance of transperineal template-guided mapping biopsy in detecting prostate cancer in the initial and repeat biopsy setting. Prostate Cancer Prostatic Dis.

[9] Eminaga O, Hinkelammert R, Abbas M (2015). Prostate cancers detected on repeat prostate biopsies show spatial distributions that differ from those detected on the initial biopsies. BJU Int.

[10] Moussa AS, Meshref A, Schoenfield L (2010). Importance of additional "extreme" anterior apical needle biopsies in the initial detection of prostate cancer. Urology.

[11] Kenigsberg AP, Tamada T, Rosenkrantz AB (2018). Multiparametric magnetic resonance imaging identifies significant apical prostate cancers. BJU Int.

[12] Seles M, Gutschi T, Mayrhofer K (2016). Sampling of the anterior apical region results in increased cancer detection and upgrading in transrectal repeat saturation biopsy of the prostate. BJU Int.

[13] Ghani KR, Dundas D, Patel U (2004). Bleeding after transrectal ultrasonography-guided prostate biopsy: a study of 7-day morbidity after a six-, eight- and 12-core biopsy protocol. BJU Int.

[14] Jones JS, Zippe CD (2003). Rectal sensation test helps avoid pain of apical prostate biopsy. J Urol.

[15] Weinreb JC, Barentsz JO, Choyke PL (2016). PI-RADS prostate imaging—reporting and data system: 2015, version 2. Eur Urol.

[16] Barentsz JO, Richenberg J, Clements R (2012). ESUR prostate MR guidelines 2012. Eur Radiol.

[17] Sauer M, Weinrich JM, Fraune C (2018). Accuracy of multiparametric MR imaging with PI-RADS V2 assessment in detecting infiltration of the neurovascular bundles prior to prostatectomy. Eur Radiol.

[18] Leyh-Bannurah SR, Kachanov M, Karakiewicz PI (2020). Combined systematic versus stand-alone multiparametric MRI-guided targeted fusion biopsy: nomogram prediction of non-organ-confined prostate cancer. World J Urol.

[19] Leyh-Bannurah SR, Kachanov M, Beyersdorff D (2018). Anterior localization of prostate cancer suspicious lesions in 1,161 patients undergoing magnetic resonance imaging/ultrasound fusion guided targeted biopsies. J Urol.

[20] Schouten MG, van der Leest M, Pokorny M (2017). Why and where do we miss significant prostate cancer with multi-parametric magnetic resonance imaging followed by magnetic resonance-guided and transrectal ultrasound-guided biopsy in biopsy-naïve men?. Eur Urol.

[21] Tu X, Liu Z, Chang T (2019). Transperineal magnetic resonance imaging-targeted biopsy may perform better than transrectal route in the detection of clinically significant prostate cancer: systematic review and meta-analysis. Clin Genitourin Cancer.

[22] Mian BM, Kaufman RP, Fisher HAG (2021). Rationale and protocol for randomized study of transrectal and transperineal prostate biopsy efficacy and complications (ProBE-PC study). Prostate Cancer Prostatic Dis.

[23] Boesen L, Norgaard N, Logager V (2017). Where do transrectal ultrasound- and magnetic resonance imaging-guided biopsies miss significant prostate cancer?. Urology.

[24] Park JS, Lee D, Koo KC (2020). The role of prostatic apex shape in voiding symptoms and urine flow: an exploratory and confirmatory study. World J Urol.

[25] Cool DW, Zhang X, Romagnoli C (2015). Evaluation of MRI-TRUS fusion versus cognitive registration accuracy for MRI-targeted, TRUS-guided prostate biopsy. AJR Am J Roentgenol.

[26] Rouviere O, Puech P, Renard-Penna R (2019). Use of prostate systematic and targeted biopsy on the basis of multiparametric MRI in biopsy-naive patients (MRI-FIRST): a prospective, multicentre, paired diagnostic study. Lancet Oncol.

[27] van der Leest M, Cornel E, Israel B (2019). Head-to-head comparison of transrectal ultrasound-guided prostate biopsy versus multiparametric prostate resonance imaging with subsequent magnetic resonance-guided biopsy in biopsy-naive men with elevated prostate-specific antigen: a large prospective multicenter clinical study. Eur Urol.

[28] Rosenkrantz AB, Verma S, Choyke P (2016). Prostate magnetic resonance imaging and magnetic resonance imaging targeted biopsy in patients with a prior negative biopsy: a consensus statement by AUA and SAR. J Urol.

