# Supplementary material for: Pan-segmental intraprostatic lesions involving mid-gland and apex of prostate (mid-apical lesions): assessing the true value of extreme apical biopsy cores
Source: World J Urol. 2022 May 2;40(7):1653–9. doi: 10.1007/s00345-022-04006-2 (PMC9236964; doi:10.1007/s00345-022-04006-2)
Supplement: Supplementary file 1 — Supplementary file1 (DOCX 469 KB) [file 345_2022_4006_MOESM1_ESM.docx]

**Supplementary Figure 1:**

**a) Visual comparison** **of mid-apical vs. exclusively apical vs. exclusively mid-gland lesions.**


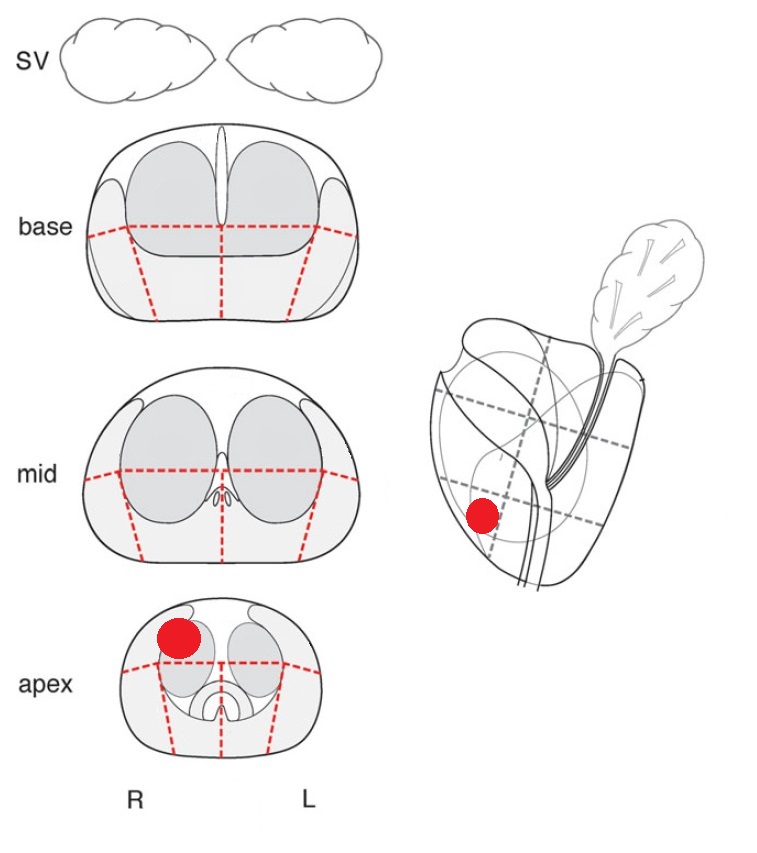

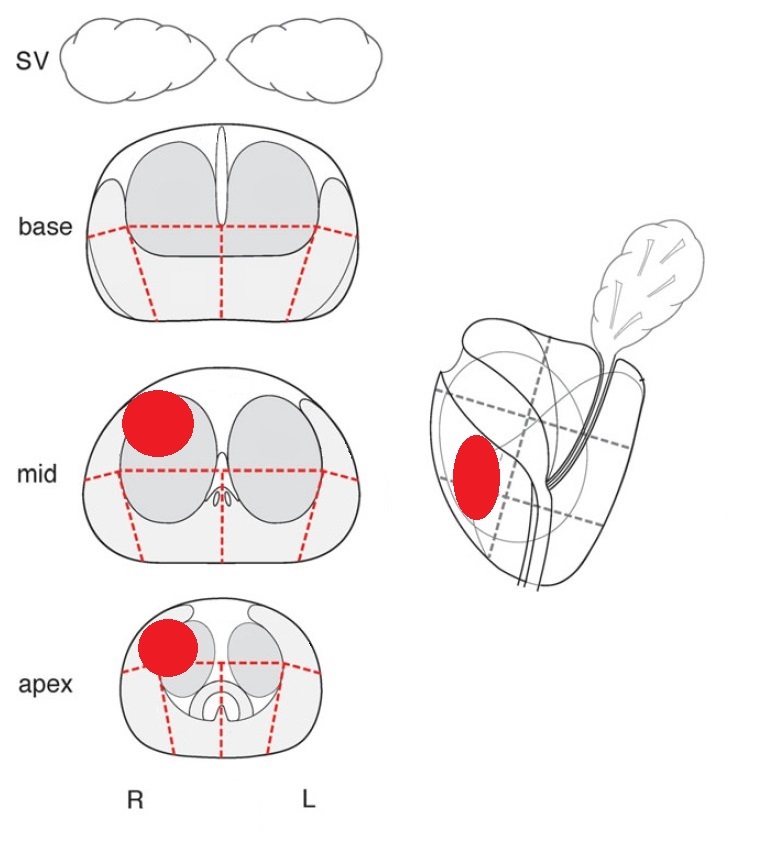


Exclusively apical lesion

Mid-apical lesion


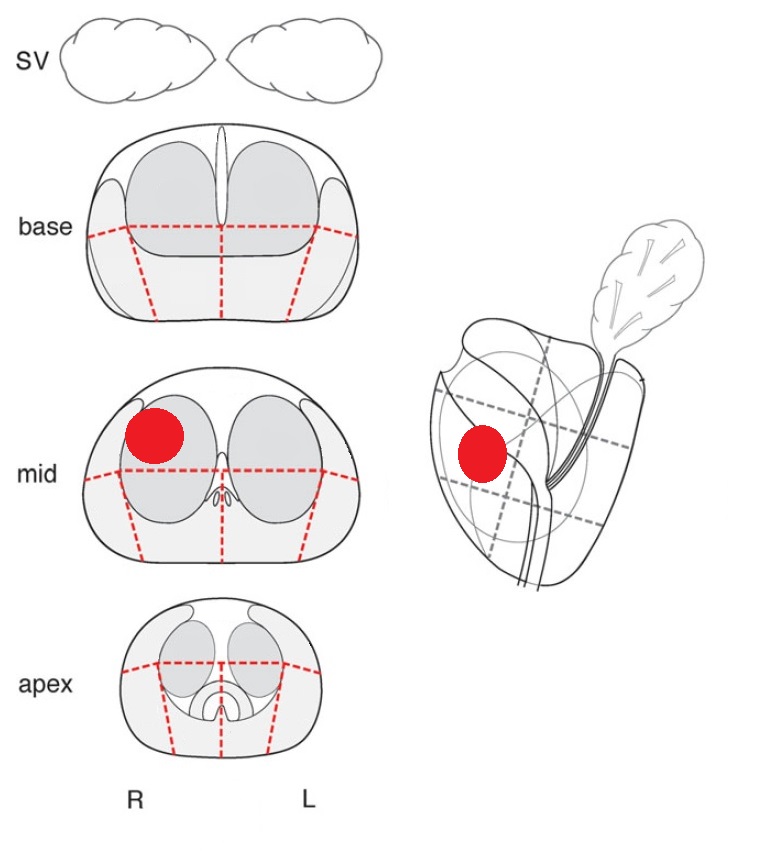


Exclusively mid-gland lesion


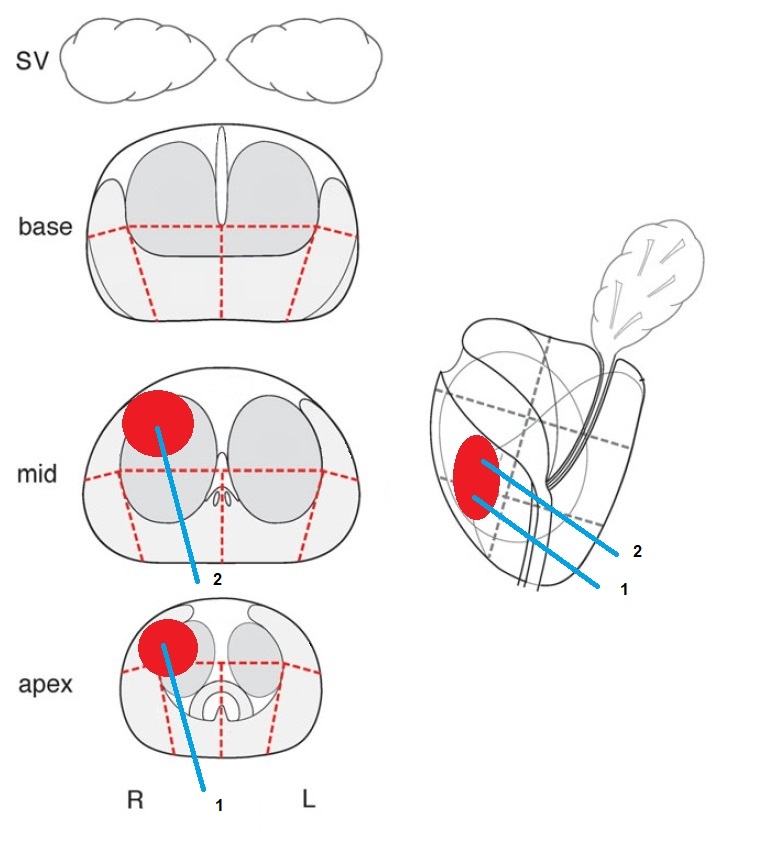
**b) Illustration of theoretical biopsy protocols implemented**


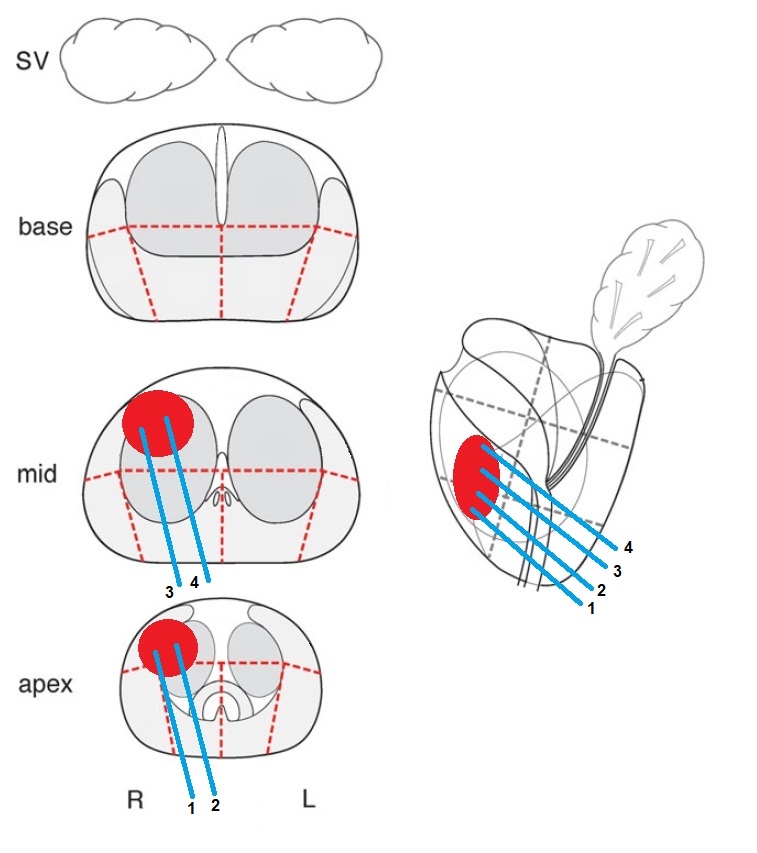


**Biopsy strategy B**

First and second targeted biopsy cores mid-gland and third and fourth cores apical

**Biopsy strategy A**

First targeted biopsy core mid-gland and second core apical
